# Zwitterionic poly(sulfobetaine methacrylate)-based hydrogel coating for drinking water distribution systems to inhibit adhesion of waterborne bacteria

**DOI:** 10.3389/fbioe.2023.1066126

**Published:** 2023-02-21

**Authors:** Olga Sójka, Henny C. van der Mei, Patrick van Rijn, Maria Cristina Gagliano

**Affiliations:** ^1^ Wetsus, European Centre of Excellence for Sustainable Water Technology, Leeuwarden, Netherlands; ^2^ Department of Biomedical Engineering, University Medical Center Groningen, University of Groningen, Groningen, Netherlands

**Keywords:** zwitterionic hydrogels, anti-adhesive coating, biofilm, bacterial adhesion, drinking water distribution

## Abstract

Presence of biofilms in drinking water distribution systems (DWDS) can be a nuisance, leading to several operational and maintenance issues (i.e., increased secondary disinfectants demand, pipe damage or increased flow resistance), and so far, no single control practice was found to be sufficiently effective. Here, we propose poly (sulfobetaine methacrylate) (P(SBMA))-based hydrogel coating application as a biofilm control strategy in DWDS. The P(SBMA) coating was synthetized through photoinitiated free radical polymerization on polydimethylsiloxane with different combinations of SBMA as a monomer, and *N, N′*-methylenebis (acrylamide) (BIS) as a cross-linker. The most stable coating in terms of its mechanical properties was obtained using 20% SBMA with a 20:1 SBMA:BIS ratio. The coating was characterized using Scanning Electron Microscopy, Energy Dispersive X-Ray Spectroscopy, and water contact angle measurements. The anti-adhesive performance of the coating was evaluated in a parallel-plate flow chamber system against adhesion of four bacterial strains representing genera commonly identified in DWDS biofilm communities, *Sphingomonas* and *Pseudomonas*. The selected strains exhibited varying adhesion behaviors in terms of attachment density and bacteria distribution on the surface. Despite these differences, after 4 h, presence of the P(SBMA)-based hydrogel coating significantly reduced the number of adhering bacteria by 97%, 94%, 98% and 99%, for *Sphingomonas* Sph5, *Sphingomonas* Sph10, *Pseudomonas extremorientalis* and *Pseudomonas aeruginosa*, respectively, compared to non-coated surfaces. These findings motivate further research into a potential application of a hydrogel anti-adhesive coating as a localized biofilm control strategy in DWDS, especially on materials known to promote excessive biofilm growth.

## 1 Introduction

Drinking water distribution systems (DWDS) are complex networks of kilometers of pipes, valves and appendages varying in material and age, and they are essential for safe transportation of water from a treatment plant to consumer taps. However, due to the variety of complex chemical, physical, and biological processes within the system, water quality often deteriorates with the distribution distance (Fish et al., 2016). In addition to pipe corrosion and scales formation, biofilm growth on DWDS surfaces is one of the main causes troubling in terms of maintenance (Makris et al., 2014). Biofilms are microbial communities consisting of cells embedded in extracellular polymeric substances that can form on any surface (Flemming et al., 2016). Their development in DWDS can lead to operational issues, such as increased secondary disinfectants demand (Adhikari et al., 2012), pipe damage through microbially induced corrosion of iron-based pipework (Wang et al., 2015), or increased flow resistance (Cowle et al., 2014). Additionally, the detachment of biofilm clusters can directly affect water quality by not only worsening its aesthetics (turbidity, color, smell) (Husband and Boxall, 2011), but also posing as a health hazard as biofilms can serve as a potential pathogen reservoir (Wingender and Flemming, 2011). Biofilm mode of life provides microorganisms in DWDS with numerous advantages, including protection from harmful compounds, mechanical stability, and nutrients scavenging and enrichment in oligotrophic conditions (Flemming and Wuertz, 2019). These properties limit the efficiency of commonly applied biofilm control strategies, such as chlorination/chloramination (Schwering et al., 2013), periodic flushing (Douterelo et al., 2013), or advanced water treatment for carbon and nutrients limitation (Park et al., 2021). Until now, no method has proven to be fully effective against already developed biofilm in DWDS environments.

It has been shown that specific surface properties play a role in biofilm formation process (Ruhal et al., 2015), and different materials utilized in DWDS were found to promote varying microbial attachment and biofilm formation. For instance, ethylene propylene diene monomer (EPDM) rubber, an elastomer commonly used for rubber-coated valves, has repeatedly shown to support excessive biofilm development compared to other commonly applied materials (Neu and Hammes, 2020). DWDS biofilm research addresses release of compounds, that either promote or inhibit microbial growth (Wang et al., 2022) or material characteristics as surface roughness (Cowle et al., 2019) that have an impact on biofilm development. To our knowledge, studies investigating the influence of other essential surface properties, i.e., hydrophilicity, charge or chemistry, playing an important role in early stages of biofilm formation, have been very limited in drinking water environment (Simões et al., 2007; Simões et al., 2010). Understanding and modification of these surface parameters can allow control over microbial adhesion and biofilm development.

One of the approaches to minimize bioadhesion through modification of surface properties is the application of hydrogel-based coatings. Hydrogels are three-dimensional cross-linked networks of hydrophilic polymers that can absorb large amounts of water (Wang et al., 2020). When deposited on a surface, in an aqueous environment, they create a hydration layer that serves as a physical and energetical barrier for bacterial adhesion, and thereby limits biofilm formation in its initial stages (Zhao et al., 2020). In the last decade, zwitterionic polymers, such as poly (sulfobetaine methacrylate) (P(SBMA)) utilized in this study, gained attention for their excellent anti-adhesive properties in the fields of biomedical (Peng et al., 2020), marine (Ventura et al., 2017), and membrane technologies (Zhu et al., 2018). Zwitterionic polymers are characterized by a net-neutral charge and a strong hydration capacity due to electrostatic attractions between ion and cation groups on the polymer pendant groups and water molecules, and are commonly utilized for preparation of hydrogels (Liu et al., 2022). UV-mediated photografting with benzophenone as a photoinitiator is one of the approaches used for hydrogel coating preparation (Keskin et al., 2018). This approach is simple as it can be performed in mild experimental conditions on a large surface area, with only pre-treatment of the surface by infusion of benzophenone (Schneider et al., 2010).

In this work, we aimed to deposit the P(SBMA) hydrogel coating with and without a cross-linker through a simple, three step photoinitiated free radical polymerization on a polydimethylsiloxane (PDMS) substrate, as a transparent substitute to elastomeric materials used in DWDS, to evaluate its effect on the adhesion of bacterial strains representing two common genera found in DWDS biofilms, *Sphingomonas* and *Pseudomonas*. Various concentrations of the monomer and cross-linker were studied to observe their effect on final coating characteristics. The coating performance against bacterial adhesion was evaluated by flowing concentrated bacterial suspensions over coated and non-coated PDMS, in a parallel-plate flow chamber set-up and enumerating the attached bacterial cells after 4 h.

## 2 Materials and methods

### 2.1 Coating of PDMS with P(SBMA)-based hydrogel

#### 2.1.1 Reagents

Polydimethylsiloxane (PDMS) samples were prepared using the Sylgard 184 elastomer kit (Dow Corning, United States) following the supplier’s instructions. The monomer 2-(methacryloyloxy) ethyl dimethyl-(3-sulfopropyl) ammonium hydroxide (95%) (sulfobetaine methacrylate; SBMA), the cross-linker *N*, *N′*-methylenebis (acrylamide) (99%) (BIS) and the photoinitiator benzophenone (BP) were purchased from Sigma-Aldrich, United States. All chemicals were used as received. Ultrapure water (18.2 MΩ, arium 611 DI water purification system; Sartorius AG, Germany) was used for preparation of monomer solutions.

#### 2.1.2 PDMS sample preparation

PDMS mixture was prepared following the manufacturer instructions by thoroughly mixing the elastomer and curing agent in a 10:1 w/w ratio. To remove bubbles from the mixture, it was degassed under vacuum (RVT360 vacuum oven, Heraeus, Germany). Approximately 0.2 g was transferred to a poly (methyl methacrylate) (PMMA) mold (20 mm × 6 mm) using a syringe. The mold was then placed in the oven and the PDMS sample was cured at 70°C overnight.

#### 2.1.3 P(SBMA) hydrogel coating synthesis on PDMS

The P(SBMA) hydrogel coating was performed through photoinitiated free radical polymerization as described previously (Keskin et al., 2018) with slight modifications (Supplementary Figure S1). For the photoinitiator infusion step, PDMS samples were incubated in a BP solution (10 wt% in acetone) for 15 min and left to dry at room temperature (RT). Aqueous SBMA solutions with or without the BIS cross-linker were prepared in different ratios (Table 1) and oxygen was removed by purging with nitrogen gas for 1 h before use. The following coating procedure was performed under a nitrogen atmosphere. Four BP infused PDMS samples were put in a quartz petri dish (Ø 100 mm) (Behr Labor-Technik, Germany) and on each of the PDMS samples, 10 µl of SBMA solution was distributed dropwise. Subsequently, a microscope glass coverslip (22 × 22 mm) was placed on top of each sample. The samples with a cover slip still in place and under nitrogen were sealed in a quartz petri dish with parafilm and irradiated for 15 min with UV in a Spectrolinker XL 1500 UV (Spectronics Corp., United States) with eight fluorescent 15-W black UV-lamps (F15T8/BLB GTE; Sylvania, United States), wavelength of 365 nm and intensity range of 2335—1385 μW/cm^2^. After the irradiation, the PDMS samples with the P(SBMA) hydrogel coating were left undisturbed overnight at RT under nitrogen. The samples were unsealed, and the glass coverslips were removed. Subsequently, the samples were washed in ultrapure water (6 ml), three times for 30 min, to remove non-polymerized monomer and cross-linker, and air-dried at RT overnight and stored in a dry state in a closed container at RT until further use. Before the adhesion experiments, the coatings were hydrated by exposing them to ultrapure water overnight.

**TABLE 1:** Composition of SBMA monomer and BIS cross-linker solutions.

	SBMA concentration [wt%]	SBMA:BIS ratio [wt]
1	20	-
2	40	-
3	20	20:1
4	20	10:1
5	40	20:1

#### 2.1.4 Scanning electron microscopy and energy-dispersive X-ray spectroscopy

The thickness and elemental composition of the coatings were analyzed using scanning electron microscopy (SEM) JEOL JSM 6480 LV (JEOL Europe B.V., Netherlands) equipped with an x-act SDD energy dispersive X-ray (EDX) spectrometer (Oxford Instruments, UK). To prepare the cross-sections of the PDMS samples with a P(SBMA) hydrogel coating, the samples were first immersed in liquid nitrogen for 10 s, and subsequently fractured in half using a pair of forceps. The fractured samples were air-dried at RT and stored in a desiccator prior to gold coating and SEM examination. The thickness of the coating was measured on three locations of three fractured samples and averaged. The elemental analysis of the cross-section of P(SBMA) hydrogel coating on PDMS samples was performed using EDX with the accelerating voltage of 15 kV and a working distance of 10 mm.

#### 2.1.5 Water contact angle measurements

The change in hydrophobicity was assessed by measuring advancing type water contact angles on P(SBMA) hydrogel-coated and non-coated PDMS samples at RT using the captive bubble technique. Contact angles were measured with the contour analysis system (OCA35, DataPhysics Instruments, Germany) by applying an air bubble of 1.5 µl on three different locations of three P(SBMA)-coated and non-coated PDMS samples. Water contact angles were calculated using the equation:

$$\theta_{W}=180{}^{\circ}\;–\;\theta_{B}$$ (1)

where θ_W_ is the water contact angle and θ_B_ is the bubble contact angle. Raw data and calculations are provided in Supplementary Data Sheet S2.

### 2.2 Bacterial adhesion to P(SBMA)-coated PDMS in a parallel plate flow chamber

#### 2.2.1 Synthetic tap water

To provide uniform chemical conditions during the adhesion experiments that are similar to those of drinking water, standardized synthetic tap water (STW) was prepared as follows: 100 mg/l NaHCO_3_, 13 mg/l MgSO_4_·7 H_2_O, 0.7 mg/l K_2_HPO_4_, 0.3 mg/l KH_2_PO_4_, 0.01 mg/l (NH_4_)_2_SO_4_, 0.01 mg/l NaCl, 0.001 mg/l FeSO_4_·7 H_2_O, 1 mg/l NaNO_3_, 27 mg/l CaSO_4_; pH = 7.50 ± 0.05 (Gomes et al., 2019).

#### 2.2.2 Bacterial strains, growth conditions and harvesting

*Sphingomonas* and *Pseudomonas* bacterial strains are commonly identified in DWDS biofilms (Douterelo et al., 2014; Liu et al., 2014; Liu et al., 2017a) and were selected for the adhesion experiments. *Sphingomonas* Sph5 and Sph10 were isolated from a fouled membrane fed with tap water and industrial wastewater, respectively, and are part of the Wetsus institute collection (De Vries et al., 2019). *Pseudomonas extremorientalis* DSM 15824 and *Pseudomonas aeruginosa* DSM 50071 were obtained from the German Collection of Microorganisms and Cell Cultures GmbH (DSMZ, Germany). The strains were first grown from a frozen Viabank beads stock (Medical Wire, UK) stored at −80°C, on R2A agar (BD Difco, United States) plates at 30°C over 72 h. For each experiment, one colony was inoculated in 10 ml R2A liquid medium (Dinkelberg Analytics GmbH, Gablingen, Germany) and grown overnight (18 h) at 30°C under agitation (150 rpm). 0.5 ml of this pre-culture was inoculated in 100 ml R2A broth, and the main culture was allowed to grow for another 24 h. Bacteria were harvested by centrifugation (5000 g, 5 min, 10°C) and washed three times with STW. Harvested bacteria were sonicated on ice for 30 s at 30 W (Vibra Cell, model VCX130; Sonics and Materials Inc., United States) to allow breaking bacterial aggregates. To prepare bacterial suspensions needed for the adhesion experiments, bacteria were counted using a Bürker-Türk counting chamber (Marienfeld, Lauda-Königshofen, Germany) and the bacterial suspension was diluted to 3 × 10^8^ cells/ml in STW.

#### 2.2.3 Bacterial adhesion tests

Bacterial adhesion on P(SBMA)-coated and non-coated PDMS was determined using a parallel plate flow chamber by flowing a bacterial suspension (3 × 10^8^ cells/ml) at RT with a shear rate of 10 s^-1^ (1.5 ml/min) for 4 h, as described previously (Busscher and Van der Mei, 2006). The parallel plate flow chamber was mounted on a phase-contrast microscope, Olympus BH-2 (Olympus Corporation, Japan), equipped with a 40x long working distance objective (Olympus ULWD-CD Plan 40 PL). Before the experiment, the system was filled with STW to remove all air bubbles. Live images were taken from the PDMS samples with or without a coating, which were placed in the bottom PMMA plate with an insert for a sample. The top plate was made of glass. Live images were acquired after summation of 15 consecutive images (time interval 1 s) to enhance the signal-to-noise ratio and to eradicate flowing bacteria from the analysis. After 4 h, images were taken on five to ten different locations on each PDMS sample. The adhering bacteria were counted manually on each imaged location and expressed per cm^2^. Raw data and calculations are provided in Supplementary Data Sheet S3.

#### 2.2.4 Statistics

To compare the number of adhered cells on uncoated and P(SBMA)-coated PDMS, Student’s t-test was used.

## 3 Results and discussion

### 3.1 P(SBMA)-based hydrogel coating and characterization

**FIGURE 1:**
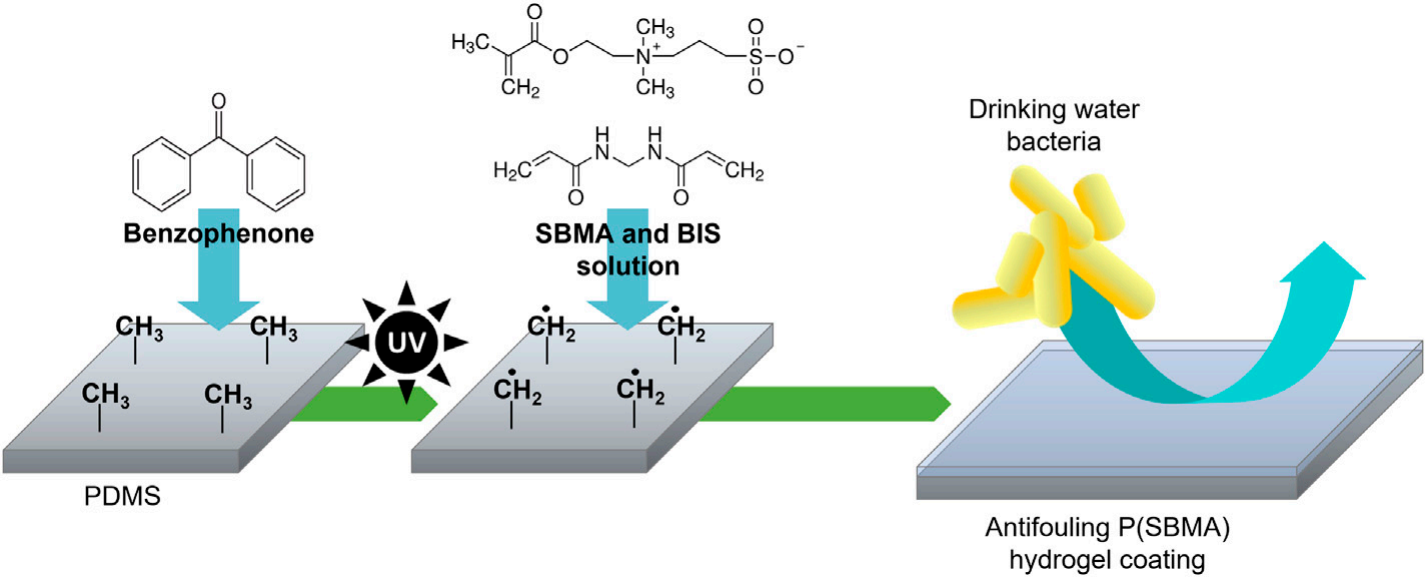
Schematic of the photografting process. UV-excitation of benzophenone infused into PDMS produces surface-bound methyl radicals that initiate polymerization of monomers present in a solution, which results in an anti-adhesive hydrogel coating covalently bound to the PDMS surface.

**FIGURE 2:**
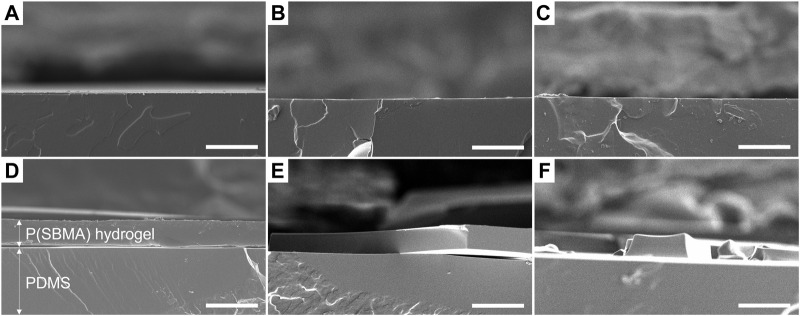
Scanning electron microscopy micrographs of PDMS cross-sections **(A)** without modification, **(B)** after coating with 20% SBMA solution without BIS addition, **(C)** 40% SBMA solution without BIS addition, **(D)** 20% SBMA solution with 20:1 ratio of SBMA to BIS, **(E)** 20% SBMA solution with 10:1 ratio of SBMA to BIS and **(F)** 40% SBMA solution with 20:1 ratio of SBMA to BIS. Scale bars are 50 μm.

P(SBMA) is a zwitterionic polymer known for its high hydrophilicity and ultralow fouling properties, which has been extensively studied in the biomedical and membrane technology fields (Zhang et al., 2018; Cascone and Lamberti, 2020). Moreover, P(SBMA) has been investigated in the context of oral drug delivery systems (Biosca et al., 2021), as it is considered non-toxic, which makes it an interesting anti-adhesive approach for DWDS. The P(SBMA)-based hydrogel coating was photografted from PDMS surface with benzophenone as a type II photoinitiator, using different monomer (SBMA) and cross-linker (BIS) concentrations (Table 1). As presented in Figure 1, UV light-induced radical formation by benzophenone that was infused into the PDMS surface, created surface-bound methyl radicals that further reacted with the monomers present in the surrounding solution initiating polymerization (Karaca Balta et al., 2015). The SEM analysis of the PDMS cross-sections showed that without the addition of the BIS cross-linker (Figure 2), the synthesis was unsuccessful, as the polymer dissolved in water in the washing steps following the polymerization, leaving the surface without any coating (Figures 2B, C). With incorporating BIS in the synthesis, the coating was present, however depending on SBMA concentration and SBMA:BIS ratio, the level of stability in terms of brittleness varied. The coatings synthetized with 20% SBMA and 10:1 SBMA:BIS ratio (Figure 2E) and 40% SBMA and 20:1 SBMA:BIS ratio (Figure 2F), were easily damaged and peeled off from a PDMS surface after drying. The most optimal outcome was achieved with 20% SBMA and 20:1 ratio of SBMA to BIS, giving a uniform and stable P(SBMA) hydrogel coating on PDMS (Figure 2D). The addition of cross-linkers in the polymerization process allows forming a covalent network that improves mechanical stability of the polymer. Selecting the right cross-linking density is essential for properties of hydrogel coatings, as it modulates not only mechanical (i.e., swelling behavior, resistance to stress and strain), but also anti-adhesive properties (i.e., stiffness) (He et al., 2019). Higher cross-linking density increases mechanical stability, however, when the cross-linking is too high, the coating becomes brittle and prone to damage (Jensen et al., 2021). The most optimal formulation of 20% SBMA with 20:1 SBMA:BIS ratio was selected for further characterization and bacterial adhesion tests.

**FIGURE 3:**
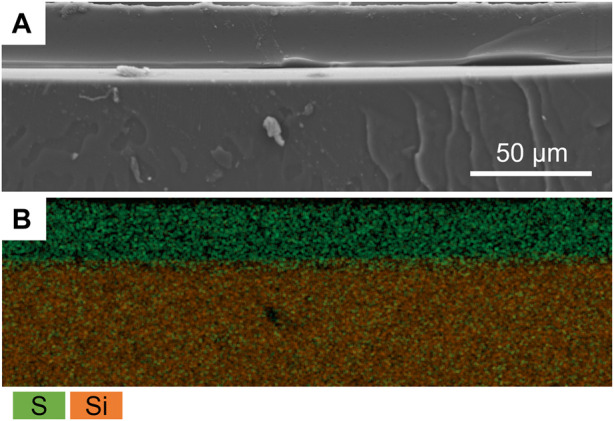
**(A)** Scanning electron microscopy image of the P(SBMA) coating (20% SBMA, 20:1 ratio SBMA to BIS) cross-section and **(B)** its energy-dispersive X-ray spectroscopy map of sulfur (green) and silica (orange) overlay representing P(SBMA) coating and PDMS support, respectively.

**FIGURE 4:**
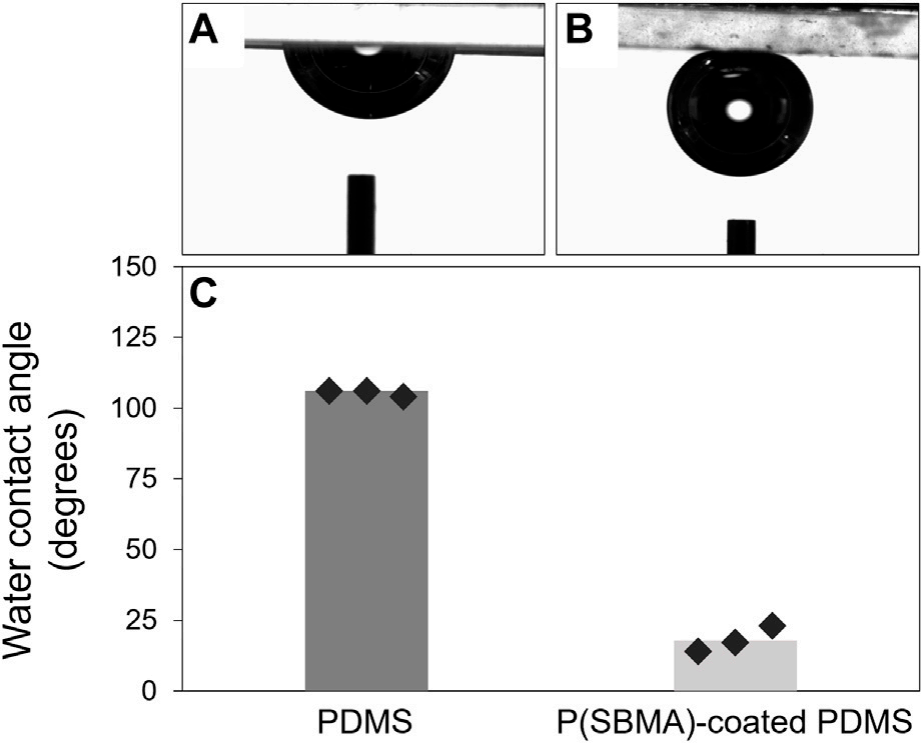
**(A, B)** Representative images of captive air bubble measurements for PDMS and P(SBMA)-coated PDMS, respectively. **(C)** Calculated water contact angles from the measured captive air bubbles in panel **(A, B)** for non-coated and P(SBMA)-coated PDMS samples. Markers represent the average value for each measured sample.

The presence of the P(SBMA) coating was further confirmed using SEM-EDX analysis by creating an elemental map of sulfur and silica as the elements representative for SBMA and PDMS, respectively, for the SEM cross-section image (Figure 3). SEM-EDX maps for other detected elements are presented in Supplementary Figure S2. The coating resulted in a homogenous layer of surface bound hydrogel of a thickness of 19 ± 4 μm in a dry state, which significantly (*p* < 0.005) increased the surface hydrophilicity by reducing the water contact angle of PDMS from 106 ± 1 to 18 ± 5°, as measured with a captive bubble method (Figure 4). When the sessile drop technique for water contact angle measurements was applied on the hydrated P(SBMA)-coated PDMS, immediate spreading occurred, and an obvious hydration layer could be observed visually (data not shown), confirming the highly hydrophilic nature and high water-absorbing capacity of P(SBMA)-based hydrogel. Surface hydrophilicity is an important parameter in predicting bacterial adhesion, as it is directly connected to surface free energy of a substratum. A more hydrophilic surface responds to higher surface free energy, which in terms of bacterial adhesion, increases the energetical demand for a bacterium to replace surface-bound water molecules (Carniello et al., 2018). A hydrogel-based coating strengthens this effect, as it absorbs and immobilizes water, creating a hydration layer that weakens the interactions between bacteria and a substratum (Chen et al., 2010). While no surface is completely resistant to biofilm development, applications of hydrophilic anti-adhesive coatings can still 1) hinder the biofilm formation, 2) promote the biofilm detachment, and/or 3) affect microbial composition of the biofilm. The presence of the hydration layer highly reduces the adhesion forces exerted by the surface, resulting in bacteria being “unaware” of their presence on the surface, and thereby, remaining in the planktonic state which does not promote biofilm phenotypic changes, such as increased EPS production (Busscher and Van der Mei, 2012; Carniello et al., 2018). However, once the initial adhesion and biofilm formation do occur, weakened interaction forces between the coated surface and those initially adhering bacteria, that also become a link between the surface and the biofilm layer formed on top of them, can lead to considerable biofilm detachment under high shear (Busscher et al., 1995; Nejadnik et al., 2008; Shave et al., 2022). In DWDS circumstances, combination of high velocity flushing, commonly used as a pipe cleaning practice (Liu et al., 2017b), and a hydrogel coating could improve the effectiveness of biofilm removal from biofilm-prone materials. Moreover, it has been recently reported, that the presence of different polymer coatings can affect biofilm bacterial composition when exposed to mixed bacterial populations (Al-Ahmad et al., 2021).

### 3.2 Adhesion tests on P(SBMA)-coated and non-coated PDMS

**FIGURE 5:**
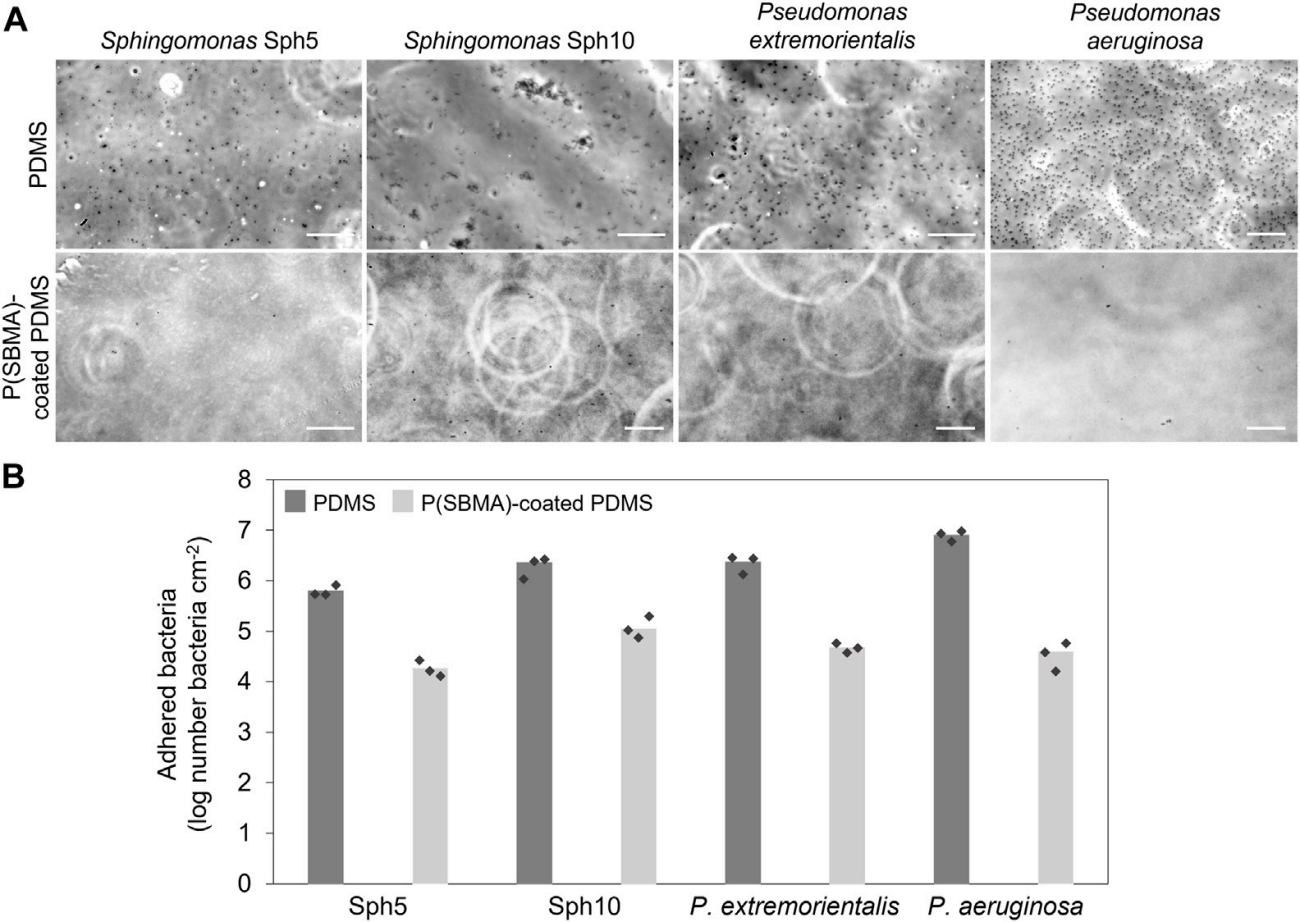
**(A)** Micrographs of four tested bacterial strains after 4 h adhesion tests on PDMS (top row) and P(SBMA)-coated PDMS (bottom row) surface. Scale bars are 20 µm. **(B)** Number of attached bacteria after 4 h adhesion on non-coated and P(SBMA)-coated PDMS. The values are averages of experiments performed on three separately coated surfaces with separately prepared bacterial cultures, indicated by the markers.

So far, P(SBMA)-based hydrogel coatings have been tested against adhesion of proteins and mammalian cells (Jensen et al., 2021; Lee et al., 2018; Leigh et al., 2019), and clinically relevant bacterial strains, such as *Staphylococcus aureus*, *Staphylococcus epidermis* and *Escherichia coli* (Shen et al., 2021; Zhang et al., 2022), proving their excellent universal anti-adhesive properties. However, the conditions in DWDS significantly vary from these in the medical field (i.e., oligotrophic environment, high water flows, spatio-temporal variations in environmental and engineering factors) (Potgieter et al., 2018). These settings select for organisms that are equipped in specific mechanisms and behaviors in terms of their survival and biofilm forming abilities, potentially different from those in clinical circumstances (Du et al., 2020). The objective of this work was to evaluate the performance of a P(SBMA)-based hydrogel coating against attachment of environmentally relevant bacteria commonly identified in DWDS biofilm communities. The four selected bacterial strains belong to the genera *Sphingomonas* and *Pseudomonas*. *Sphingomonas* spp. can persist in oligotrophic conditions (Ohta et al., 2004) and are recognized as primary surface colonizers and a dominating species in various fouling phenomena, including those on spiral wound membranes (De Vries et al., 2019), and in membrane bioreactors (Huang et al., 2008). *Pseudomonas* spp. represent a ubiquitous group of environmental bacteria, occupying several niches, including DWDS, and the presence of *P. aeruginosa*, as an opportunistic pathogen, is of hygienic relevance in DWDS biofilms (Moritz et al., 2010). Bacterial suspensions were flown for 4 h over non-coated and P(SBMA)-coated PDMS surfaces, after which the images of adhered bacteria were taken and evaluated (Figure 5). Selected bacteria exhibited varying attachment behaviors, even within the same genus. While *Sphingomonas* Sph5, *P. extremorientalis* and *P. aeruginosa* adhered as single cells, even though still varying in density, *Sphingomonas* Sph10 auto-aggregated in the liquid phase and adhered as a mix of single cells and multicellular clusters. This corresponds to initial Sph10 characterization reported by De Vries and colleagues (2019), where after 24 h, up to 100% of the bacteria in the liquid culture formed aggregates. *P. aeruginosa* adhered in the highest numbers to PDMS. The presence of the P(SBMA) coating significantly (*p* < 0.05) reduced the number of adhering bacteria compared to non-coated PDMS by 97% ± 2%, 94% ± 3%, 98% ± 1%, and 99% ± 1% for Sph5, Sph10, *P. extremorientalis* and *P. aeruginosa*, respectively (Figure 5B), and: Lower anti-adhesive efficiency of the coating against Sph10 compared to other strains, could be caused by the fact that it adheres in the form of aggregates. Bright field microscopy allows the visualization of bacteria within a two-dimensional plane. While the other three strains that adhered as single cells could be accurately quantified on the collected images, for Sph10, the three-dimensional nature of the aggregates might have led to underestimation of adhered bacteria number on the non-coated PDMS, and thereby, the anti-adhesive efficiency of the coating. The versatility of the coating’s anti-adhesive mechanism is especially important since the microbial community in DWDS is a dynamic and environment-dependent system, that represents a variety of microbial species (Potgieter et al., 2018), likely exhibiting a wide range of adhesion and biofilm forming behaviors. Moreover, the fact that the P(SBMA) coating can significantly reduce the adhesion of bacterial aggregates is of value, as in DWDS, microbial concentration in bulk water is low and detached biofilm clusters, that are also more resistant to disinfectants than planktonic bacteria, could potentially be the main initiators of downstream biofilm development (Wang et al., 2021).

### 3.3 P(SBMA)-based hydrogel coating as an alternative biofilm control strategy in the DWDS

Biofilm formation and detachment in DWDS lead to several operational and maintenance issues for water companies, whose aim is to deliver microbiologically safe drinking water to consumers (Simões and Simões, 2013). Biofilm development is driven by a variety of complex processes along the distribution network and so far, no single control practice (i.e., periodical flushing, disinfectants dosing) proved to be sufficiently effective against biofilm formation and persistence (Liu et al., 2017a). Elements utilized in DWDS, such as pipes, appendages, and valves, are manufactured from multiple materials of whose the properties affect biofilm formation to a varying degree. With this study, we successfully introduced the concept of surface properties modification with the hydrogel-based anti-adhesive coating against drinking water bacteria as a localized biofilm control strategy.

P(SBMA)-based hydrogel coating is a safe, effective, and universal anti-adhesive solution. In terms of application in DWDS, it provides an interesting alternative that could target biofilm hotspots, characterized either by local higher temperatures, long stagnation periods and dead-ends (Simunič et al., 2020), and/or sections with elements manufactured from materials especially prone to biofilm development, such as EPDM on rubber-coated valves (Neu and Hammes, 2020). Rubber-based materials such as EPDM (M-class) rubber are generally used for production of sealing elements in fittings and other products in contact with drinking water, which organic additives, however, can migrate in water, thus are highly susceptible to biofilm formation (Szczotko et al., 2016). In particular, EPDM has showed to promote microbial growth of many different microorganisms, including protozoa (Waines et al., 2011), and to stimulate also the attachment of pathogens such as *P. aeruginosa* and *Legionella*, which can then cause secondary contamination of water (Moritz et al., 2010; Szczotko et al., 2016). In this study, the P(SBMA) coating was synthetized on a transparent PDMS surface for the sake of real-time monitoring of adhesion and non-destructive quantification of adhering bacteria, but generally the UV-mediated free radical polymerization with benzophenone infusion step can be applied to other elastomeric materials of various dimensions, including EPDM rubber (Yagci et al., 2010).

Concluding, PDMS was successfully coated with P(SBMA)-based hydrogel using photografting. The P(SBMA) coating showed excellent anti-adhesive properties against *Sphingomonas* and *Pseudomonas* after 4 h, limiting their attachment by up to 99%. These results bring a lot of promise for the potential applicability of the coating in DWDS biofilm hotspots, and on elements utilizing materials that are known to promote excessive biofilm growth. However, further investigation is needed to evaluate the coating performance and stability in more representative conditions (i.e., extended testing period, exposure to DWDS hydraulic conditions and mixed microbial community), and to optimize the coating process and coating’s mechanical properties to ensure a long-lasting durability on representative materials, so essential in the proposed field of application.

## Data Availability

The original contributions presented in the study are included in the article/Supplementary Material, further inquiries can be directed to the corresponding author.
