# Supplementary material for: Zwitterionic poly(sulfobetaine methacrylate)-based hydrogel coating for drinking water distribution systems to inhibit adhesion of waterborne bacteria
Source: Front Bioeng Biotechnol. 2023 Feb 21;11:1066126. doi: 10.3389/fbioe.2023.1066126 (PMC9989184; doi:10.3389/fbioe.2023.1066126)
Supplement: Supplementary file 2 [file DataSheet1.PDF]

SUPPLEMENTARY MATERIAL

Zwitterionic poly(sulfobetaine methacrylate)-based hydrogel coating for drinking water distribution systems to inhibit adhesion of waterborne bacteria

Olga Sójka[1,2], Henny C. van der Mei[2], Patrick van Rijn[2], Maria Cristina Gagliano[1]

[1]Wetsus, European Centre of Excellence for Sustainable Water Technology, Leeuwarden, The Netherlands

[2] Department of Biomedical Engineering, University Medical Center Groningen, University of Groningen, Groningen, The Netherlands

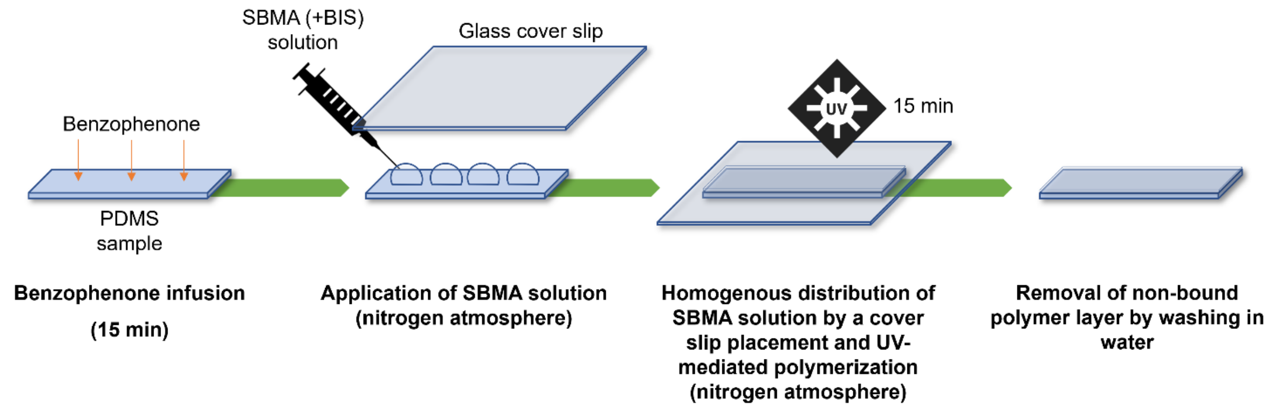

Supplementary Figure 1. Schematic of the procedure of PDMS coating with P(SBMA) hydrogel.

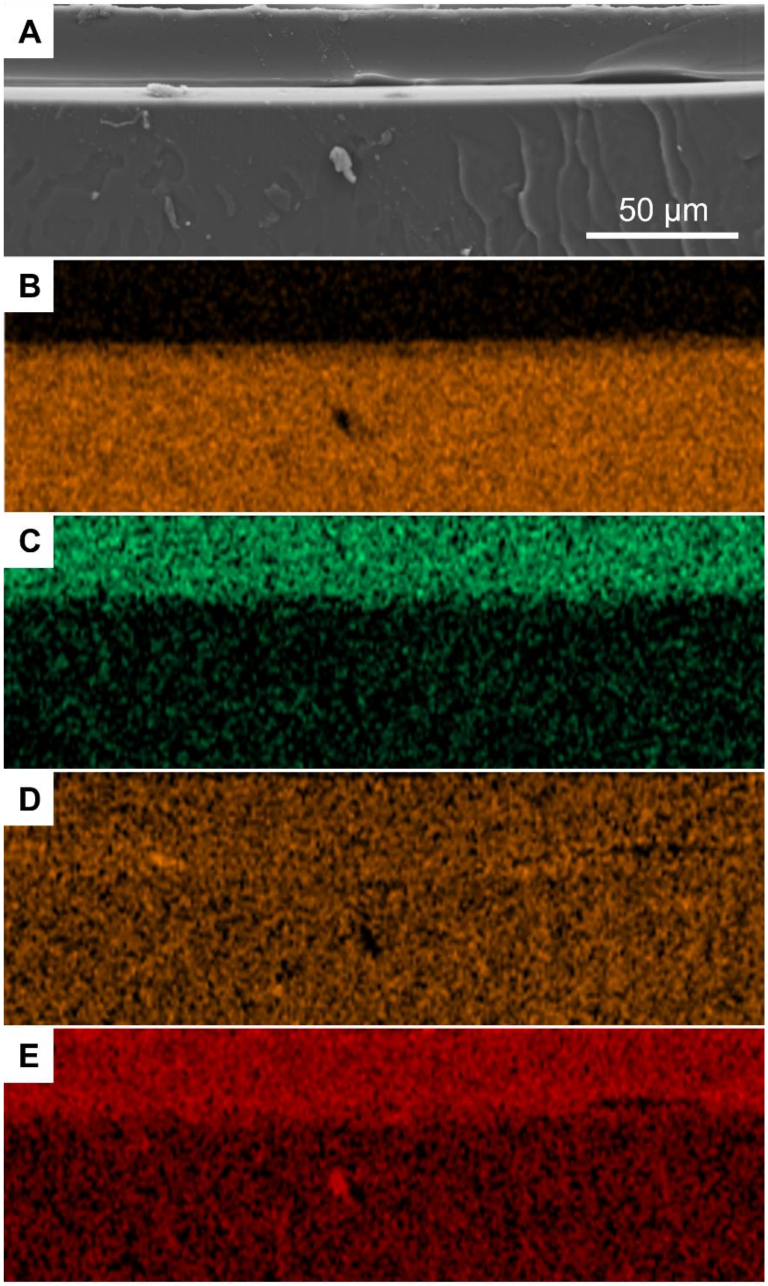

Supplementary Figure 2. (A) Scanning electron microscopy image of the P(SBMA) coating (20% SBMA, 20:1 ratio SBMA to BIS) on PDMS cross-section and its energy-dispersive X-ray spectrometry maps of **(B)** silica, **(C)** sulphur, **(D)** oxygen and **(E)** carbon.
